# Physiological epicotyl dormancy and recalcitrant storage behaviour in seeds of two tropical Fabaceae (subfamily Caesalpinioideae) species

**DOI:** 10.1093/aobpla/pls044

**Published:** 2012-12-21

**Authors:** K. M. G. Gehan Jayasuriya, Asanga S. T. B. Wijetunga, Jerry M. Baskin, Carol C. Baskin

**Affiliations:** 1 Department of Botany, University of Peradeniya, Peradeniya, Sri Lanka; 2 Department of Biological Sciences, Rajarata University of Sri Lanka, Mihinthale, Sri Lanka; 3 Department of Biology, University of Kentucky, Lexington, KY, USA; 4 Department of Plant and Soil Sciences, University of Kentucky, Lexington, KY, USA

## Abstract

**Background and aims:**

Physiological epicotyl dormancy in which the epicotyl elongates inside the seed before the shoot emerges has been reported for only a few tropical rainforest species, all of which are trees that produce recalcitrant seeds. In studies on seeds of Fabaceae in Sri Lanka, we observed a considerable time delay in shoot emergence following root emergence in seeds of the introduced caesalpinioid legumes *Brownea coccinea* and *Cynometra cauliflora.* Thus, our aim was to determine if seeds of these two tropical rainforest trees have physiological epicotyl dormancy, and also if they are recalcitrant, i.e. desiccation sensitive.

**Methodology:**

Fresh seeds were (i) dried to various moisture levels, and (ii) stored at −1 and 5 °C to determine loss (or not) of viability and thus type of seed storage behaviour (orthodox, recalcitrant or intermediate). To identify the kind of dormancy, we tested the effect of scarification on imbibition and monitored radicle emergence and epicotyl growth (inside the seed) and emergence.

**Principal results:**

Fresh seeds of both species had high moisture content (MC): 50 % for *C. cauliflora* and 30 % for *B. coccinea*. Further, all seeds of *C. cauliflora* and the majority of those of *B. coccinea* lost viability when dried to 15 % MC; most seeds of both species also lost viability during storage at −1 or 5 °C. Intact seeds of both species were water permeable, and radicles emerged in a high percentage of them in <30 days. However, shoot emergence lagged behind root emergence by 77 ± 14 days in *B. coccinea* and by 38 ± 4 days in *C. cauliflora*. Further, plumule growth inside seeds of *C. cauliflora* began almost immediately after radicle emergence but not until ∼30–35 days in *B. coccinea* seeds.

**Conclusions:**

Seeds of both species are recalcitrant and have physiological epicotyl dormancy. The kind of physiological epicotyl dormancy in seeds of *C. cauliflora* has not been described previously; the formula is *C*_nd_ (root)− (epicotyl).

## Abstract (precis)

We report epicotyl dormancy of two tropical Ceasalpinioid species; *Browneacoccinea, Cynometracauliflora*. Heretofore, epicotyl dormancy has been reported in only one Ceasalpinioid species.The kind of dormancy in seeds of *C. cauliflora*was described with the new formula C_nd_(root)–C^p′^_1b_

## Introduction

The most common kind of epicotyl dormancy described to date is epicotyl morphophysiological dormancy (MPD) that occurs in some seeds with an underdeveloped embryo (Baskin and Baskin 1998, 2004). However, epicotyl dormancy has also been identified in a few species whose seeds have a fully developed embryo: *Quercus alba*, *Quercus prinus* (Farmer 1977), *Quercus ilicifolia* (Allen and Farmer 1977), *Platonia insignis* (Maurão and Beltrati 1995; Carvalho *et al*. 1998), *Chionanthus retusus* (Chien *et al*. 2004), *Calophyllum brasiliensis, Lecythis ampla* (Flores 1996), *Garcinia kola* (Agyili *et al*. 2007) and *Humboldtia laurifolia* (Jayasuriya *et al*. 2010). Thus, this kind of epicotyl dormancy cannot be classified as a level (*sensu* Baskin and Baskin 2004) of MPD. Baskin and Baskin (1998) referred to the acorns of the white oaks as having a specialized kind of epicotyl dormancy, thus distinguishing it from epicotyl MPD. Recently, Jayasuriya *et al*. (2010) reported still another kind of epicotyl dormancy in seeds of the Sri Lankan tropical rainforest understorey tree *H. laurifolia* (Fabaceae, subfamily Caesalpinioideae), in which the shoot needs to grow to a considerable length inside the seed before it can emerge. They called this kind of dormancy physiological epicotyl dormancy and used the formula *C*_nd_ (root)−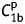 (epicotyl) to describe it (see below).

Seeds of all species thus far reported as having a fully developed embryo and epicotyl dormancy are recalcitrant except for those of *C. retusus* (Jayasuriya *et al*. 2010); in addition, all of them are woody. Species known to have epicotyl dormancy belong to families in different clades (APG 2009). In our studies on seed dormancy in Sri Lankan Fabaceae, we observed a delay in shoot emergence in two other species of Fabaceae, subfamily Caesalpinioideae, namely *Brownea coccinea* and *Cynometra cauliflora*, which suggests that seeds of these two species may also have physiological epicotyl dormancy. Thus, the aim of our research was to confirm (or not) that seeds of these two species have physiological epicotyl dormancy and also to determine whether the seeds are desiccation sensitive in storage behaviour. *Brownea coccinea* and *C. cauliflora* are important ornamental species in South and Southeast Asian countries (Rudd 1991), and in Manaus, Brazil, the latter species is planted as a street tree (Nazario *et al*. 2008). Thus, in addition to being of interest to basic seed scientists, the information generated in this research may be important for the propagation and seed storage of these ornamentally and ecologically important species. Information about seed dormancy and storage behaviour for tropical species in Fabaceae is not commonly available in the literature.

Available literature on both tropical and temperate species suggests that seeds of most Fabaceae species have physical dormancy (Baskin and Baskin 1998). However, some species produce seeds that are non-dormant (Ramdeo 1970; Teketay 1998; Kumar *et al*. 2007; Sautu *et al*. 2007) or have combinational (Sautu *et al*. 2007) or physiological (Baskin and Baskin 1998; Sautu *et al*. 2007) dormancy. Moreover, embryos of Fabaceae are fully developed (Martin 1946), and thus neither morphological dormancy nor MPD is present in seeds of members of this family.

Seeds of most Fabaceae have orthodox storage behaviour (Dickie and Pritchard 2002). However, there are also species in this family with intermediate and recalcitrant seed storage behaviour (Dickie and Pritchard 2002; Oliveira-Silveira *et al*. 2005; Saba *et al*. 2008). As is true for seed dormancy, information on seed storage behaviour of tropical Fabaceae species is limited. Thus, our research adds to the knowledge base on seed dormancy and storage behaviour of tropical Fabaceae species.

Ours is the first detailed study on seed germination of *Cynometra* species. Whitman (1972) stated that seeds of *C. cauliflora* germinate readily without any treatment. However, he did not give any information about the actual germination percentage of this species and also defined germination as radicle emergence, which tells us nothing about shoot emergence. Ng (1992) reported that seeds of *C. cauliflora* planted outdoors in West Malaysia germinated (growth and emergence of embryo to form a seedling) in 19–59 days and those of *C. elmeri* in 40–150 days; seedlings from fruits of the latter species emerged in 75–105 days. Kubitzki and Ziburski (1994) obtained 60 % germination (three of five seeds) in *C. spruceana* var. *phaselocarpa* under ‘standard conditions’ (not described); the number of days to germination (mean ± SD) was 81.7 ± 30.7. However, the authors did not define germination. Seeds of this species submerged under water for 60 days germinated to 80 % (four of five seeds). In a study by Moreira and Moreira (1996), the number of days to 50 % germination (radicle emergence) in *Cynometra bauhiniifolia* was 28. Seeds germinated between Days 20 and 52 after sowing, and final germination was 90 %. Nothing was said about time to shoot emergence. Nazario *et al*. (2008) showed that >80 % of the *C. bauhiniifolia* seeds germinated when they were incubated within the first month after collection. However, they did not document shoot emergence. Further, Nazario *et al*. (2008) showed that seeds lost viability when dried to a moisture content (MC) <28.6 %, which suggests that seeds of *C. bauhinifolia* have recalcitrant storage behaviour. We are not aware of any information on seed germination or storage behaviour of *Brownea* species.

## Methods

### Study organisms

*Brownea coccinea* Jacq. and *C. cauliflora* L. belong to the plant family Fabaceae, subfamily Caesalpinioideae, tribe Datarieae (Mabberley 2008). *Brownea coccinea* is a native understorey tree in tropical rainforests in South America (Klitgaard 1991; Macmillan 1993; Kenny 2006) and was introduced into Sri Lanka in the 19th century as an ornamental plant (Rudd 1991). The genus consists of ∼12 species that are restricted to the New World tropics; its geographic range extends from Costa Rica and the West Indies to Peru (Mabberley 2008). *Cynometra cauliflora* was introduced as an ornamental tree to Sri Lanka in the 18th century from Malaysia, where it is native (Rudd 1991; Macmillan 1993; Mabberley 2008). The species is also a rainforest understorey tree in its native range (Macmillan 1993). *Cynometra* consists of ∼85 species distributed in both the Old World and New World tropics (Mabberley 2008).

### Collection of seeds

Mature seeds were collected from numerous haphazardly selected roadside and ornamentally grown trees of *C. cauliflora* in the wet zone (Ambalangoda, Peradeniya and Kandy) of Sri Lanka and those of *B. coccinea* from five trees in the Royal Botanic Gardens, Peradeniya, Sri Lanka. Seeds of both species were collected during December 2008 to February 2009 and during June to August 2009, put in labelled polythene bags and transported to the Department of Botany, University of Peradeniya, Sri Lanka. Experiments were initiated within 2 weeks from the collection date.

### Seed storage behaviour

The purpose of these experiments was to determine whether seeds are orthodox, recalcitrant or intermediate in storage behaviour. Orthodox seeds can be dried to 2–5 % MC and stored at sub-zero temperatures (optimum c. −18 °C) without losing viability. Recalcitrant seeds tend to have high initial seed MC (≥15 % fresh mass basis) compared with orthodox seeds, and they lose viability when dried to <15 % (fresh mass basis) MC. Further, recalcitrant seeds of tropical origin lose viability when they are stored at low temperatures (<10 °C). The third seed storage category, intermediate, was created to accommodate seeds of the relatively small percentage of species that do not fit into either of the other two categories. Seeds with intermediate storage behaviour are less tolerant of drying than orthodox seeds (c. 6–12 % MC), and their optimum storage temperature is >0 °C. Realistically, however, seed storage behaviour can be viewed as a continuum rather than as discrete categories (Hong and Ellis 1996; Berjak and Pammenter 2002).

#### Initial seed MC

The mass of 15 seeds of each species was measured individually with a digital analytical balance to the nearest 0.0001 g. Seeds were placed in glass aliquots and oven-dried to constant mass at 110 °C. Initial seed MC was calculated on a fresh mass basis.

#### Effect of drying on seed viability

Three samples of three replicates containing 15 seeds each were weighed to the nearest 0.0001 g using a digital analytical balance and air-dried at ambient laboratory temperatures (c. 25 °C) in open 9-cm-diameter Petri dishes until they reached 30, 20 or 10 % MC. Seed MC was calculated using the decrease in mass of the air-dried seeds. The calculated initial seed MC for fresh seeds of *C. cauliflora* and *B. coccinea* was 50 and 30 %, respectively. Targeted weight of the seeds at desired MC was calculated using the following formula, suggested by Hong and Ellis (1996):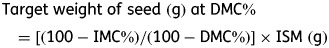 where DMC is the desired MC, IMC is the initial MC and ISM is the initial seed mass. When seeds reached the target mass, they were tested for viability by incubating them on filter papers moistened with 100 p.p.m. gibberellic acid (Wako Pure Chemical Industries Ltd, Chuo-ku, Osaka, Japan) in 9-cm-diameter Petri dishes at ambient laboratory temperature (c. 25 °C) and light conditions (artificial fluorescent room light for 9 h per day plus diffused sunlight through windows). Seeds were monitored for germination (radicle emergence, indicating viability) at 2-day intervals for 30 days, at which time non-germinated seeds were cut open to check the viability of the embryo. The presence of a firm white embryo indicated that the seed was viable, and a soft, grey embryo indicated that the seed was non-viable.

#### Effect of low-temperature storage on seed viability

Four samples of three replicates of at least 10 seeds from each species were stored in dry 9-cm-diameter Petri dishes, which were placed in sealed ziplock bags to prevent moisture loss and stored at 5 °C and at −1 °C or at 5 °C for 1 and 2 months. Retrieved seeds were incubated on filter papers moistened with 100 p.p.m. gibberellic acid in 9-cm-diameter Petri dishes at ambient laboratory temperature (c*.* 25 °C) and light conditions. Seeds were monitored for germination (radicle emergence, indicating viability) at 2-day intervals for 30 days, at which time non-germinated seeds were cut open to check the viability of embryos.

### Kind of seed dormancy

The purpose of these experiments was to determine to which of the five dormancy classes (*sensu* Baskin and Baskin 1998, 2004) seeds of *B. coccinea* and *C. cauliflora* belong. If seeds have physical dormancy (PY), intact seeds will not imbibe water, while scarified seeds will imbibe water and germinate in <30 days when they are kept on water-moistened filter papers. If seeds have physiological dormancy (PD), they will imbibe water but take >30 days to germinate. If non-scarified seeds do not imbibe, whereas scarified seeds do imbibe but take >30 days to germinate, the seeds have combinational dormancy (PY+PD). If intact seeds imbibe and take <30 days to germinate, they are non-dormant. Seeds of Fabaceae have a fully developed embryo and thus cannot have either morphological dormancy or MPD (Baskin and Baskin 2004; Finch-Savage and Leubner-Metzger 2006). However, if there is a significant time delay between radicle emergence and shoot emergence, seeds have physiological epicotyl dormancy (Jayasuriya *et al*. 2010), a subclass of PD (J. M. Baskin and C. C. Baskin, unpubl. data).

#### Imbibition of water by seeds

Two samples of 15 fresh untreated (intact-fresh) and manually scarified (individually with a razor blade) seeds of each species were weighed individually with a digital analytical balance to the nearest 0.0001 g and then placed in Petri dishes on filter papers moistened with water at ambient laboratory conditions. Seeds were retrieved at the time intervals shown in Fig. 3, blotted dry, reweighed and returned to the Petri dishes. The experiment was continued for 30 days or until all the seeds germinated (radicle emergence), whichever occurred first.

#### Seed germination

Four replicates of 15 fresh untreated (intact) seeds of each species were placed on water-moistened filter papers in 9-cm-diameter Petri dishes and incubated at ambient laboratory conditions. Seeds were monitored for germination (radicle emergence) at 3-day intervals for 3 months.

### Time taken for root and shoot emergence

The purpose of these experiments was to determine whether seeds of *C. cauliflora* and *B. coccinea* have physiological epicotyl dormancy. Fifteen seeds of each species were selected haphazardly and individually labelled. Seeds were placed on water-moistened filter papers in 9-cm-diameter Petri dishes and incubated at ambient laboratory temperature and light conditions. They were monitored at 2-day intervals for radicle and shoot emergence, and dates of radicle protrusion and of shoot emergence were recorded for each seed.

### Root and shoot development

The purpose of these experiments was to monitor root emergence and shoot growth (inside the seeds) and emergence. Twenty samples of five seeds of each species were selected haphazardly and incubated on water-moistened filter papers in 9-cm-diameter Petri dishes at ambient temperature and light conditions. Each sample was retrieved at the intervals shown in Fig. 5. Seeds were cut into halves and radicle and plumule lengths measured with a ruler to the nearest millimetre.

### Data analysis

Experiments were arranged in a completely randomized design. Data collected on the effect of storage on viability, the effect of drying on viability and initial seed germination experiments were analysed using the one-way analysis of variance procedure. Means were separated using Duncan's multiple range procedure. Final imbibition data of untreated and manually scarified seeds were analysed using pooled *t*-tests for each species separately. The difference between time taken for radicle emergence and time taken for shoot emergence following radicle emergence was tested using paired *t*-tests for each species. Discrete data were arc-sine transformed prior to analysis. Four-parameter Weibull sigmoid regression curves were fitted to the data on the effect of drying on germination and also for root and shoot development measurements.

## Results

### Seed storage behaviour

#### Initial seed MC

Fresh mass of *C. cauliflora* and *B. coccinea* seeds was 4.3 ± 1.3 and 19.5 ± 4.6 g, respectively, and their dry mass was 2.2 ± 0.8 and 13.7 ± 3.5 g, respectively. The initial MC (fresh mass basis) of *C. cauliflora* and *B. coccinea* seeds was 49.8 ± 3 and 29.8 ± 4 %, respectively.

#### Effect of drying on seed viability

**Fig. 1:**
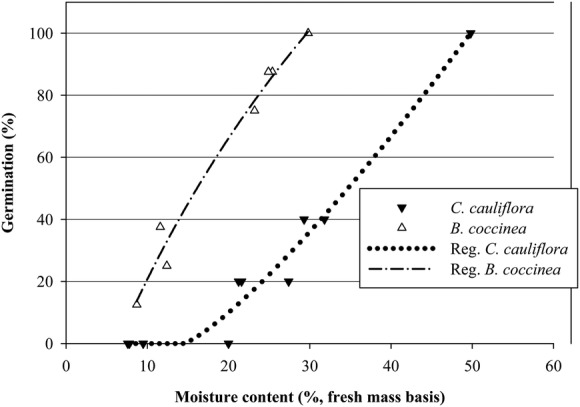
**Weibull four-parameter sigmoid regression relationship between seed MC and % germination (= viability) of *B. coccinea* and of *C. cauliflora* seeds.** Reg., regression.

The germination percentage of seeds of both species decreased with a decrease in seed MC (Fig. 1). All non-germinated seeds rotted within a few days. There were highly significant Weibull four-parameter sigmoid relationships between seed germination percentage and MC for both species. *Cynometra cauliflora* seeds lost viability completely when dried to <15 % MC. However, ∼35 % of the *B. coccinea* seeds remained viable even when they were dried to <15 % MC. Drying increased the time taken for seeds of *B. coccinea* to germinate (data not shown).

#### Effect of low-temperature storage on seed viability

**Fig. 2:**
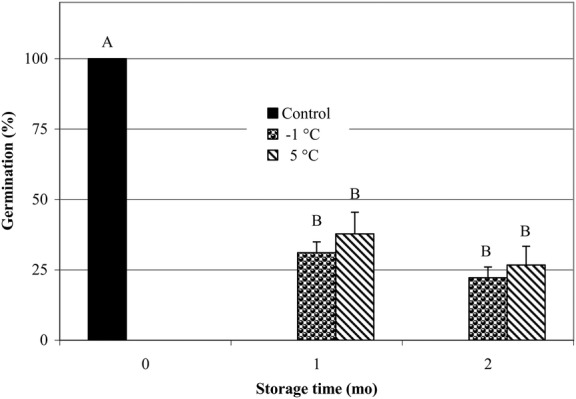
**Germination of *B. coccinea* seeds after wet storage at −1 or 5** °**C for 0, 1 or 2 months.** Different letters indicate significant differences between all treatments. Error bars = +1 SEM.

None of the *C. cauliflora* seeds germinated after they had been stored wet for 1 or 2 months at −1 or 5 °C (data not shown). However, 31 and 22 % of the *B. coccinea* seeds germinated after 1 and 2 months of wet storage at −1 °C, respectively, and 38 and 27 % of them germinated after 1 and 2 months of wet storage at 5 °C, respectively (Fig. 2). Non-germinated seeds rotted within 2–3 days.

### Kind of seed dormancy

#### Imbibition of seeds

**Fig. 3:**
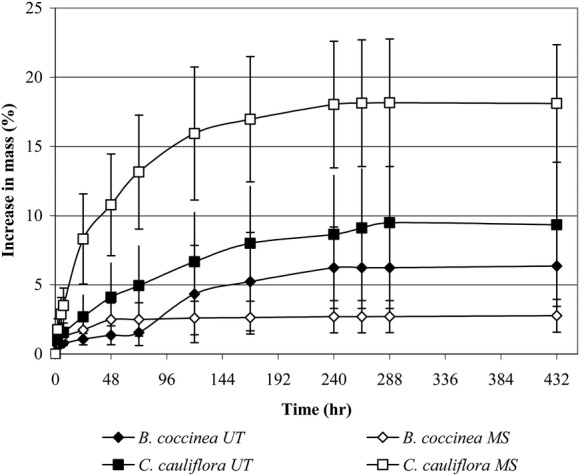
**Imbibition of manually scarified and intact (untreated) seeds of *B. coccinea* and *C. cauliflora***. Error bars = ±1 SEM. UT, untreated; MS, manually scarified.

Mass of manually scarified seeds and of untreated intact seeds of both species increased <25 % during imbibition (Fig. 3). There were no significant differences between manually scarified seeds vs. intact seeds of either species.

#### Seed germination

Seeds of both species have the hypogeal type of germination. Ninety and 100 % of untreated *B. coccinea* and *C. cauliflora* seeds*,* respectively, germinated (radicle emergence) within 30 days. Seeds of *C. cauliflora* germinated faster than those of *B. coccinea*, and *T*_50_ was 9.5 and 13.5 days, respectively.

### Time taken for root and shoot emergence

**Fig. 4:**
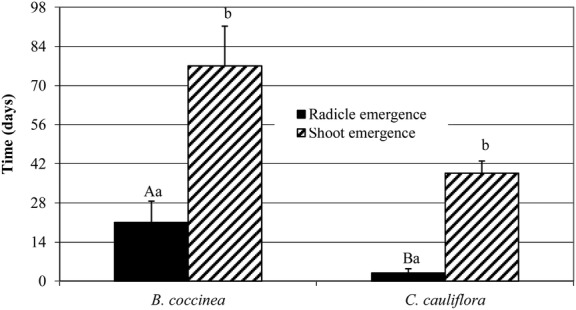
**Days to radicle emergence (from date of incubation) and to shoot emergence (from date of root emergence) of untreated intact seeds of *B. coccinea* and *C. cauliflora* at ambient laboratory temperature and light conditions.** Different uppercase letters indicate significant differences between germination (radicle emergence) of seeds of the two species, and different lowercase letters indicate significant difference between shoot emergence and radicle emergence in the same species. Error bars = +1 SEM.

Radicle emergence occurred in 21 ± 8 and 3 ± 2 days for *B. coccinea* and *C. cauliflora*, respectively, and shoot emergence in 77 ± 14 and 38 ± 4 days, respectively, after radicle emergence (Fig. 4).

### Root and shoot development

**Fig. 5:**
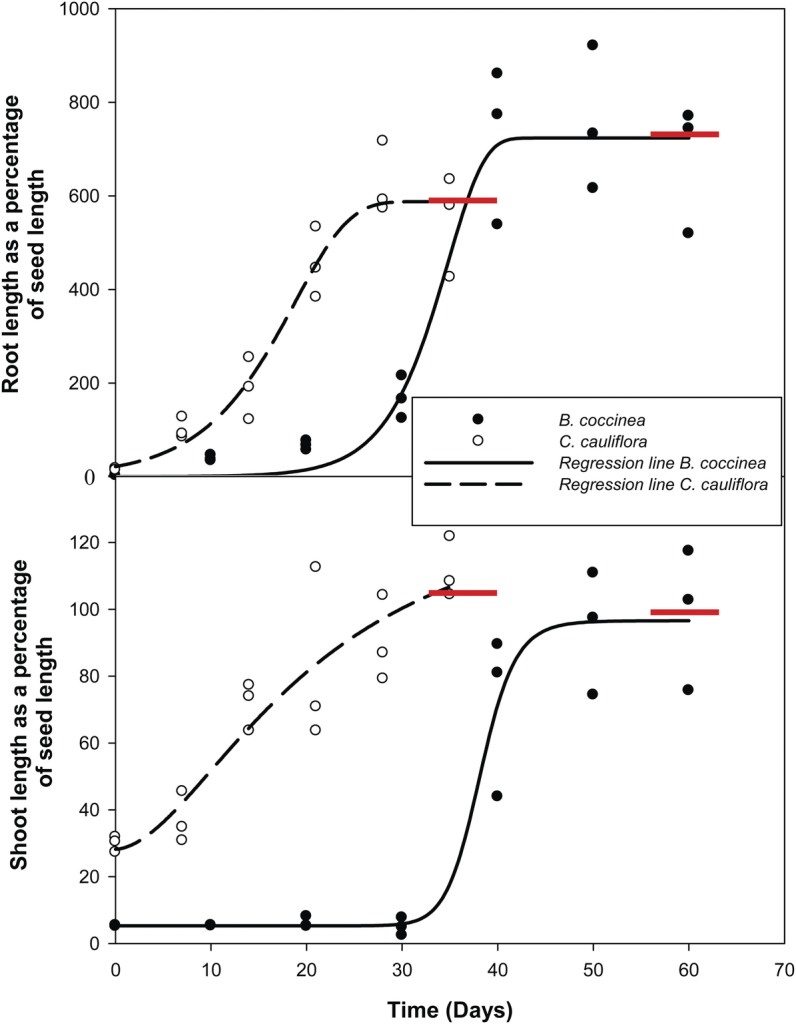
**Second-degree polynomial regression lines fitted to root length as a percentage of seed length (top panel) and to shoot length as a percentage of seed length (bottom panel) increase with time for seeds of *B. coccinea* and *C. cauliflora* incubated at ambient laboratory temperature and light conditions.** Red horizontal lines indicate times of shoot emergence. Time 0 indicates the commencement of radicle emergence.

At the time of radicle emergence in *B. coccinea*, the shoot axis of the embryo (SAE) was ∼5 % of the length of the seed. The SAE grew very little during the first 30 days after radicle emergence (Fig. 5). However, after 40 days the SAE was ∼70 % of seed length. Within 65–85 days following radicle emergence, the shoot emerged from the seed. By this time, the length of the SAE was ∼98 % of the seed length (Fig. 5). In *C. cauliflora*, the SAE started to grow as soon as the radicle emerged from the seed. However, the rate of growth was very low. As a result of this low growth rate, shoot emergence in *C. cauliflora* was delayed ∼38 days from radicle emergence. At the time of radicle emergence, the SAE was ∼30 % of the length of the seed, and 14 days after radicle emergence it was ∼70 % of seed length. After 28 days, the length of the SAE had increased to ∼95 % of seed length, and shoot emergence occurred after ∼35–40 days, by which time the shoot was ∼104 % of seed length.

## Discussion

Freshly mature seeds of both *B. coccinea* and *C. cauliflora* had >15 % seed MC, which suggested that they might have recalcitrant storage bahaviour. Recalcitrant behaviour was confirmed by dry storage and low-temperature storage experiments. Weibull sigmoidal curves fitted to the seed drying data for both species showed that seeds lost viability as they dried. The two MC–viability curves for *B. coccinea* and *C. caulifolia* resemble the typical desiccation tolerance curves for intermediate and recalcitrant species, respectively (Hong and Ellis 1996). Further, Hong and Ellis (1996) suggested that recalcitrant seeds cannot be stored dry at <10 °C or under hermetical conditions at −20 °C for 3 months. None of the seeds of *C. cauliflora* germinated after 1 month storage at −1 or 5 °C, which shows that seeds of this species cannot survive under the condition mentioned by Hong and Ellis (1996). Thus, it can be concluded that seeds of *C. caulifolia* are recalcitrant in their storage behaviour. However, ≥20 % of the *B. coccinea* seeds retained viability even when they were dried to 10 % MC, and 22 and 26 % of them survived after 2 months of storage at −1 and 5 °C, respectively. Thus, it appears that while most seeds of *B. coccinea* are recalcitrant, a portion of them have intermediate seed storage behaviour. In any case, seeds of both species are desiccation sensitive.

In the imbibition experiment, there were no significant differences between mass increase in manually scarified and untreated intact seeds of either species. Thus, seeds of both species have a water-permeable seed coat, and unlike many species of Fabaceae (Baskin and Baskin 1998) they do not have PY. When only radicle protrusion is used as the criterion for germination, seeds of both species are non-dormant; radicle protrusion occurs in ≤30 days (see Sautu *et al*. 2007). Although radicle emergence in *B. coccinea* and *C. cauliflora* occurred within 21 ± 8 and 3 ± 2 days, respectively (from the beginning of incubation), shoot emergence was delayed until ∼77 ± 14 and 38 ± 4 days, respectively, after radicle protrusion. Thus, seeds of both species have epicotyl dormancy. Generally, seeds with PY need to dry to <20 % MC for the seed or fruit coat to become water impermeable (Qu *et al*. 2010). Thus, it is not at all surprising that seeds of *C. cauliflora* and *B. coccinea* with 50 and 30 % MC, respectively, did not have PY. Furthermore, the seeds of *Brownea* and *Cynometra* are overgrown, and thus their testa is undifferentiated (Corner 1951). Consequently, the seed coat does not have a palisade layer and is water permeable.

The plumule did not begin to elongate in seeds of *B. coccinea* until ∼30–35 days following root emergence. Then, it started to grow gradually, and shoots emerged from the seeds after about another 35–40 days. Thus, *B. coccinea* seeds require only a period of warm stratification to overcome epicotyl dormancy, and we can conclude that the epicotyl of this species has non-deep PD. This phenomenon is similar to that of *H. laurifolia*, in which the epicotyl did not begin to grow until 37 days after the radicle emerged (Jayasuriya *et al*. 2010). Jayasuriya *et al*. (2010) suggested the formula *C*_nd_ (root)−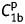 (epicotyl) to describe the epicotyl dormancy in seeds of *H. laurifolia*. Using symbols of Nikolaeva and Baskin and Baskin (see Baskin and Baskin 2008), except for superscripts p′ and p, which are new (see below), *C*_nd_ (root) indicates that the root is physiologically (*C*) non-dormant (subscript nd) and 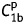 that the epicotyl (superscript p) is physiologically (*C*) dormant, requiring a warm period (subscript 1b) to break dormancy (Jayasuriya *et al*. 2010). The same formula describes epicotyl dormancy in seeds of *B. coccinea*.

Delay in shoot emergence following root emergence also occurs in seeds of *C. cauliflora*. However, unlike the situation in *H. laurifolia* and *B. coccinea* the shoot axis of *C. cauliflora* begins to grow within the seed soon after the root emerges, i.e. little or no delay. The shoot grew gradually and emerged from the seed after ∼38 ± 4 days following radicle protrusion. To complete germination (root and shoot emergence out of the seed), it took only ∼42 ± 5 days under ambient laboratory conditions (Fig. 5). We suggest the following formula to describe physiological epicotyl dormancy in *C. cauliflora*: *C*_nd_ (root)−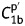 (epicotyl). Thus, the only difference in this formula and the one for *Humboldtia* and *Brownea* is superscript p′ instead of p. That is, superscript p and p′ represent plumule growth within the seed with and without a delay, respectively, following root emergence.

In certain respects, dormancy in seeds of *B. coccinea* and *C. cauliflora* is similar to that in seeds of *H. laurifolia* (Jayasuriya *et al*. 2010), *Q. alba, Q. prinus* (Farmer 1977) and *Q. ilicifolia* (Allen and Farmer 1977). The radicle is non-dormant [*C*_nd_ (root)] in all of them. However, in seeds of *B. coccinea, H. laurifolia, Q. alba, Q. prinus* and *Q. ilicifolia* the plumule or epicotyl undergoes a no-growth stage following radicle emergence, in contrast to *C. cauliflora* in which the plumule or epicotyl begins to grow as soon as the radicle emerges. Thus, in seeds of *C. cauliflora* the delay in shoot emergence is due to the slow growth rate of the plumule or epicotyl, while in the other species a delay in shoot emergence additionally is due to a period of no growth of the plumule or epicotyl (*B. coccinea*, *H. laurifolia*) or only to a period of no growth (*Quercus* spp.).

Thus far, physiological epicotyl dormancy has been reported in five flowering plant families: Clusiaceae, *P. insignis* (Maurão and Beltrati 1995; Carvalho *et al*. 1998) and *G. kola* (Agyili *et al*. 2007); Fabaceae, *B. coccinea, C. cauliflora* (current study) and *H. laurifolia* (Jayasuriya *et al*. 2010); Fagaceae, *Q. alba, Q. prinus* (Farmer 1977) and *Q. ilicifolia* (Allen and Farmer 1977); Lecythidaceae, *L. ampla* (Flores 1996); and Oleaceae, *C. retusus* (Chien *et al*. 2004). Clusiaceae, Fabaceae and Fagaceae belong to the eurosids I clade, Oleaceae to euasterids I clade and Lecythidaceae to basal euasterids (APG 2009). Clusiaceae, Fabaceae and Fagaceae are closely related to each other, whereas Oleaceae and Lecythidaceae are not closely related to each other and distantly related to these three eurosid families (APG 2009). Thus, physiological epicotyl dormancy may have evolved independently in recalcitrant seeds in the eurosid I clade and in the orthodox seeds in the euasterid I clade and recalcitrant seeds of basal euasterids. However, more examples are needed to document (or not) any trend in the phylogeny of physiological epicotyl dormancy.

The three tropical Fabaceae species known to have physiological epicotyl dormancy are understorey trees in rainforests [*H. laurifolia* (Ashton *et al*. 1997), *Brownea coccinea* (Kenny 2006) and *C. cauliflora* (Macmillan 1993)]. Further, the epicotyl dormancy in these three species differs from epicotyl dormancy in the canopy tree *P. insignis* (Clusiaceae), the only other tropical species reported to have physiological epicotyl dormancy for which we can provide a dormancy formula at the present time; in *P. insignis*, the epicotyl is deeply dormant [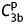 (shoot)].

The three Fabaceae species with physiological epicotyl dormancy and recalcitrant seeds are in the tribe Datarieae, subfamily Caesalpinioideae (Rudd 1991). *Anthonotha* (Breteler 2010), *Browneopsis* (Klitgaard 1991), *Crudia* (Herendeen and Dilcher 1990), *Microberlinia* (Newbery *et al.* 2004), *Eperua* (Cowan 1975) and *Intsia* (Thaman *et al.* 2006) are some of the Datarieae genera that also occur in tropical rainforests. However, seeds of none of these genera are reported to have physiological epicotyl dormancy. Seeds of *Browneopsis* (Klitgaard 1991), *Microberlinia* (Norghauer and Newbery 2011) and *Eperua* (Fogart 1992) have been reported to be non-dormant, those of *Intsia* to have PY (Ng 1992) and those of *Crudia* to be dormant (PY or PD) (da Silva *et al.* 1989; Ng 1992). Other caesalpinioid species producing orthodox seeds mainly have physical dormancy (Baskin and Baskin 1998).

The ecological significance of epicotyl dormancy in tropical recalcitrant species is not yet clear. Normally, recalcitrant seeds have a rapid germination strategy (both root and shoot emergence) that allows them to escape high seed pathogen and predation risks, and they occur in mesic environments (Kermode and Finch-Savage 2002). In seeds with physiological epicotyl dormancy, there is a considerable delay between radicle and shoot emergence. Thus, seeds with only the radicle emerged stay on the forest floor for a relatively long period of time. Seeds of temperate *Quercus* species with epicotyl dormancy require cold stratification for shoot emergence (Allen and Farmer 1977; Farmer 1977), whereas tropical species require warm stratification (Carvalho *et al*. 1998; Jayasuriya *et al*. 2010) for shoot emergence. *Quercus* species use this strategy to time completion of the germination event (shoot emergence) in the spring, while the roots emerge soon after dispersal of the acorns in autumn (Farmer 1977; Thoreau 2000). We know of no field studies on the physiological epicotyl dormancy in seeds of tropical species that can be used to evaluate the ecological significance of this kind of dormancy. However, our observations led us to hypothesize that these species use this strategy to time the germination event when the emerging shoot will be exposed to suitable light conditions for seedling establishment. Early radicle emergence may be essential for the recalcitrant seeds to maintain viability in the shoot axis and for the seed to escape predators and pathogens.

## Conclusions and forward look

Seeds of *B. coccinea* and *C. caulifora* are desiccation sensitive to varying degrees and have physiological epicotyl dormancy. The kind of dormancy in seeds of *C. cauliflora* has not been reported previously, and it can be described with the formula *C*_nd_ (root) − 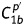 (epicotyl). Physiological epicotyl dormancy may be common in tropical species with recalcitrant seeds. However, seed dormancy studies are needed on more species, especially non-pioneer species in tropical rainforests, to reveal the evolutionary and ecological significance of physiological epicotyl dormancy.

## Sources of funding

This research was funded by personal funds of K.M.G.G.J. and A.S.T.B.W.

## Contributions by the authors

K.M.G.G.J. and A.S.T.B.W. initiated the development of the concept, designed and conducted the experiments and wrote the first version of the manuscript. J.M.B. and C.C.B. assisted in further development of the concept, interpretation of results and revision of several drafts of the manuscript.

## Conflicts of interest statement

None declared.
